# 47th Nordic Lung Congress - Oslo 2015

**DOI:** 10.3402/ecrj.v2.28179

**Published:** 2015-06-04

## Abstracts

## ASTHMA 01

### The correlation between airway hyperresponsiveness and maximal oxygen uptake in untrained adult asthmatics

#### Kerstin Kiis, Louise Lindhardt Toennesen, Morten Hostrup Nielsen and Vibeke Backer
kerstinkiis@outlook.com
Bispebjerg University Hospital, København, Denmark

#### 

*Objective*: To investigate the relationship between airway hyperresponsiveness (AHR) and maximal oxygen consumption (VO2-max) in a group of sedentary non-obese asthma patients. *Method*: As part of an ongoing clinical trial of lifestyle interventions in asthma, 39 sedentary non-obese asthma patients were examined at two separate test days. At day 1, spirometry was performed as well as a bronchial provocation test with mannitol to assess AHR. Levels of fractional exhaled nitrogen oxide (FeNO) were measured as a marker of eosinophilic airway inflammation, and asthma control was assessed using the Asthma Control Questionnaire (ACQ). At day 2, a skin prick test was performed, as well as a dual-energy X-ray absorptiometry (DXA) scan and an incremental bicycle ergometer test to exhaustion for determination of VO2-max. *Results*: There was no correlation between VO2-max and the degree of AHR to mannitol (rp=0.04, *p*=0.84) or FeNO (rp=0.10, *p*=0.55). There were no difference in VO2-max between patients who used inhaled corticosteroids and those who did not. Patients’ fat percent correlated positive with VO2-max (rp=−0.36, *p*<0.05). *Conclusion*: The degree of AHR to mannitol does not predict VO2-max in sedentary non-obese asthma patients. However, further investigations among a larger and more heterogeneous group of asthma patients should be performed to further elucidate this correlation.

Keyword: *asthma/allergy*

## 02

### Poorer asthma control is associated with high levels of physical activity in young females but not males

#### Ludvig Lövström, Margareta Emtner, Kjell Alving, Christer Janson and Andrei Malinovschi

Andrei.Malinovschi@medsci.uu.se
Uppsala University, Uppsala, Sweden

#### 

Studies on the levels of physical activity in asthma patients compared with controls report varying results. We have recently published that high versus moderate levels of physical activity were associated with higher prevalence of wheezing, especially in females (1). Therefore, we studied levels of physical activity in young asthmatic and healthy subjects, and the relation between physical activity levels and asthma control. A total of 405 subjects with physician-diagnosed asthma and 118 controls (10–34 years) answered questions concerning frequency and duration of physical activity. Subjects with asthma performed spirometry, methacholine challenges, and exhaled NO measurements and answered Asthma Control Test (ACT). Asthma patients were physically active more frequently (*p*=0.01) and for longer durations (*p*=0.002) than controls. Not well-controlled asthma (ACT<20) was more prevalent in the highly versus moderately physically active group, seen to both frequency (40.2% vs. 23.9%, *p*=0.001) and duration (39.0% vs. 21.7%, *p*<0.001). This was consistent only for females (*p*<0.05). Frequently versus moderately active females were 4.9 (2.5–9.8) more likely to have ACT<20 while no such effect was found in men [OR 1.1 (0.6–2.2)] (*p*-value for interaction with gender=0.003). No differences in FeNO or methacholine reactivity were found between moderately and highly physically active females with asthma. In conclusion, young patients with asthma were more active than controls. High levels of physical activity were associated with poor asthma control in females, but not males, and this appears unrelated to airway inflammation or responsiveness. Further studies are needed to better understand the mechanism behind the poorer control of asthma in this group.

**Reference**

1. Jerning C, Martinander E, Bjerg A, Ekerljung L, Franklin KA, Järvholm B, et al. Asthma and physical activity – a population based study results from the Swedish GA(2)LEN survey. Respir Med. 2013; 107: 1651–8.

## 03

### Low-volume/high-intensity aerobic interval training improves maximal oxygen consumption in untrained adult asthmatics

#### Nick Meier, Louise Tønnesen, Morten Hostrup and Vibeke Backer
nickmeier@live.dk
Bispebjerg Hospital, Copenhagen N, Denmark

#### 

*Objective*: The main aim of this study was to evaluate the effect of a low-volume/high-intensity training intervention on maximal oxygen consumption (VO2max) in a group of untrained adult asthma patients. *Method*: Five and thirteen untrained men and women, respectively, with a respiratory physician's diagnose of asthma and an Asthma Quality of Life score (ACQ)≥1 were included and randomized into either a training group (TG, *n*=8) or a control group (CG, *n*=10). The training intervention consisted of 8 weeks of 10–20–30 training, consisting of low-, moderate-, and high-speed cycling on stationary indoor cycles (<30, <60 and >90% of maximal intensity) for 30, 20 and 10 sec, respectably, in 5 min intervals, interspersed by 2 min of recovery. The patients exercised for 20 min, three times a week. *Results*: One patient in the TG dropped out during the intervention period due to an intercurrent respiratory tract infection, and one patient in the CG failed to follow the control protocol and was therefore excluded from the analysis. The low-volume/high-intensity interval training resulted in significant increases in VO2max, when compared to control (*P*<0.0001). The percentage increase in VO2max in the TG was +11±9% (mean, SD), reflecting increases in VO2max from 34.5±4.5 mL/kg to 38.4±5.8 mL/kg. *Conclusion*: A 10–20–30 exercise regime with low-volume/high-intensity training is well tolerated in untrained adult asthma patients and promotes significant aerobic fitness improvements within 8 weeks of intervention.

Keywords: *asthma/allergy*; *rehabilitation*

## 04

### Varied combination of breathing disorders among women referred to methacholine bronchial challenge test for confirmation of asthma

#### Maria Ragnarsdottir

mariara@internet.is
Landspitali National and University Hospital, Reykjavik, Iceland

#### 

*Objective*: To investigate the prevalence of hyperventilation syndrome, rapid breathing, and thoracic dominant type of respiratory movement during quiet and voluntary deep breathing among women suspected to have asthma. *Methods*: Subjects were 20 women undergoing bronchial challenge test. Their height and weight were measured, and they answered the Nijmegen Questionnaire together with standardized questions regarding diseases or trauma that could adversely affect chest movements. Respiratory movements were measured in supine position for 1 min during quiet and voluntary deep breathing using the Respiratory Movement Measuring Instrument. *Results*: Thirteen subjects had positive bronchial challenge test (65%), eleven (55%) had more than 20 Nijmegen scores, thereof had eight (40%) ≥23 scores, four subjects (20%) had rapid breathing, three had thoracic dominant type of breathing during quiet breathing and eleven during voluntary deep breathing. One fourth of the study group had neither positive methacholine bronchial challenge test nor Nijmegen Questionnaire score. *Conclusion*: The diverse combination of symptoms among women referred to undergo methacholine bronchial challenge test to confirm the diagnosis of asthma found in this study suggests that thorough assessment of their breathing is needed to give individual targeted treatment, avoid using unnecessary medication, and reduce anxiety of the unknown.

Keyword: *asthma/allergy*

## 05

### Airway inflammatory cells in induced sputum in athletes: a comparative study of athletes with and without asthma and healthy non-athletes

#### Julie Stang ^1^, Liv Ingunn Bjoner Sikkeland^2^, Trine Stensrud^1^ and Kai-Håkon Carlsen^1,2^

julie.stang@nih.no

^1^Norwegian School of Sport Sciences, Oslo, Norway; ^2^University of Oslo, Norway.

#### 

*Introduction*: Increased percentage of sputum neutrophils has been reported in half-marathon runners both at baseline and after exercise. In swimmers and cold-air athletes, the number of training hours/week correlates with percentage of sputum neutrophils. The aim of the present study was to compare baseline sputum inflammatory cells in healthy and asthmatic endurance athletes. We also wanted to assess if there was an association between bronchial hyperresponsiveness (BHR) and sputum inflammatory cells. *Methods*: In this cross-sectional study, 21 athletes with self-reported asthma (♀=7), 19 healthy athletes (♀=5) and 22 healthy non-athletes (controls) (♀=11) underwent allergy skin prick test, methacholine bronchial challenge, and induced sputum. All athletes were top athletes in swimming (*n*=20) and cross-country skiing (*n*=20). Mean±SD training hours/week was 18.3 (±4.9). Medication prior to testing was withheld according to current guidelines. *Results*: Mean percentage of sputum neutrophils, eosinophils, macrophages or bronchial epithelial cells did not differ when comparing the healthy controls with healthy and asthmatic athletes. Sixty-seven percent of the asthmatic athletes had BHR (PD20<8 µmol methacholine) as compared to 58% of healthy athletes and 32% of non-athletes. No differences in sputum inflammatory cells were observed between subjects with and without BHR, and the degree of BHR did not correlate with sputum inflammatory cells. *Conclusion*: Airway inflammation was not present at baseline in neither asthmatic nor healthy swimmers and cross-country skiers, suggesting that the previously reported acute inflammatory responses resulting from heavy exercise may be reversible. BHR was increased in both healthy and asthmatic athletes, but was not related to airway inflammation.

Keyword: *asthma/allergy*

## 06

### Increased decline in pulmonary function among employees in Norwegian smelters reporting work-related asthma-like symptoms

#### Vidar Søyseth ^1^, Helle Johnsen^2^, Paul K. Henneberger^3^ and Johny Kongerud^4^

vidar.soyseth@medisin.uio.no

^1^Akershus University Hospital, Lørenskog, Norway; ^2^Institute of Occupational Health, Oslo, Norway; ^3^National Institute of Occupational Safety and Health, Morgantown, USA; ^4^Oslo University Hospital, Norway

#### 

*Introduction*: We have investigated the association between work-related asthma-like symptoms (WASTH) and annual decline in pulmonary function among the employees in 18 Norwegian smelters. *Methods*: All the employees were invited to participate in a 5-year longitudinal study. Spirometry was performed annually in each employee. WASTH was defined as the combination of dyspnea and wheezing improving on rest days or vacation in an individual who had no asthma previously. Information about smoking and occupational status was obtained from a questionnaire. The data were analyzed using linear mixed model. *Results*: The total number of follow-ups was 12,996 in 3,084 employees. The crude annual decline in forced expiratory volume in 1 sec (dFEV1) and forced vital capacity (dFVC) was 32.9 (95% confidence interval: 30.5 to 35.3) mL/year and 40.9 (37.8 to 43.9) mL/year, respectively. The similar results in subjects reporting WASTH was 45.9 (26.8 to 65.0) and 68.8 (45.6 to 92.0) mL/year, respectively. Stratified analyses showed that the association between dFEV1 as well as dFVC and WASTH was modified by history of familial asthma (*p*=0.014 and *p*=0.0008, respectively). The overall difference in dFEV1 and dFVC between employee reporting WASTH and not reporting WASTH was 14.6 mL/year (95% CI: 2.1 to 27.1) and 28.8 mL/year (95% CI: 13.1 to 44.5), respectively. *Conclusion*: WASTH was associated with an increased annual decline in FEV1 as well as FVC indicating a restrictive pattern.

Keyword: *occupational/environmental medicine*

## COPD 07

### Prevalence and characteristics of the asthma-COPD overlap syndrome – or the asthma-like COPD phenotype – in a COPD high-risk population

#### Camilla Boslev Bårnes, Peter Kjeldgaard, Mia Nielsen and Charlotte Suppli Ulrik

boslev@gmail.com
Hvidovre Hospital, Hvidovre, Denmark

#### 

*Objective*: Our aim was to characterize prevalence and characteristics of asthma COPD overlap syndrome (ACOS), based on different definitions, in a cohort of adults at high risk of COPD. *Method*: General practitioners (*n*=241) consecutively recruited subjects ≥35 years old, with tobacco exposure, at least one respiratory symptom, and no previous diagnosis of obstructive lung disease. ACOS was defined as chronic airflow obstruction, that is, post-bronchodilator FEV1/FVC ratio<0.70, combined with either wheeze, significant BD reversibility, or both reversibility and lung function impairment, that is, FEV1<80%pred. *Results*: Of the 3.875 (50% females, mean age 57 years) subjects included in the study, 700 (18.1%) were diagnosed with COPD, defined as symptom(s), current or previous tobacco exposure, and a post-bronchodilator FEV1/FVC ratio<0.70. The prevalence of ACOS among patients diagnosed with COPD was 27% (wheeze), 16% (BD reversibility), and 14% (BD reversibility and FEV1<80%pred), respectively (*p*<0.001). ACOS patients had significant lower post-BD FEV1/FVC ratio compared to COPD only patients (*p*=0.02). ACOS patients with self-reported wheeze (*n*=190) had lower FEV1%pred compared to COPD only patients (66% vs. 73%; *p*<0.0001), whereas no difference was found in the level of FEV1%pred between ACOS and COPD only patients when the two other definitions were applied. Irrespective of the ACOS-definition, no significant difference in life-time tobacco exposure was found between ACOS and COPD only patients. *Conclusion*: In subjects with a new diagnosis of COPD, the prevalence of ACOS, or asthma-like COPD, is high, but the prevalence and characteristics of patients classified as having ACOS differs substantially depending on the applied diagnostic criteria.

Keyword: *COPD*

## 08

### Assessment of Type D personality in stable COPD patients detects psychological distress and vulnerability for acute exacerbation

#### Gunnar Einvik ^1,2^, Angelica Marianne Berg^1^, Anke Neukamm^1,2^, Vidar Søyseth^1,2^, Natalia Kononova^1,2^, Arne Didrik Høiseth^1,2^, Torbjørn Omland^1,2^ and Toril Dammen^2^

guneinvik@gmail.com

^1^Akershus University Hospital, Lørenskog, Norway; ^2^University of Oslo, Norway

#### 

*Introduction*: Psychological factors, such as anxiety and depression, may influence the clinical progression of chronic obstructive pulmonary disease (COPD). Type D personality is another psychological factor characterized by the stable tendency toward both negative affectivity and social inhibition. The prevalence of Type D personality among COPD patients, its relation to COPD-severity, and influence on acute exacerbations are not established. *Methods*: A total of 179 patients with stable COPD, attending the outpatient department at Akershus University Hospital, completed the DS14 questionnaire, Hospital Anxiety and Depression Scale (HADS), and a standard evaluation of pulmonary function. Hospitalizations for AECOPD were recorded. *Results*: A total of 27 patients (15%) had Type D personality. Type D personality was associated with significantly higher scores of anxiety and depression, while the COPD severity was similar as for non-Type D. During a median follow-up of 2.7 years, 58 patients (33%) were hospitalized due to AECOPD. The crude ratios for AECOPD were 9.8 and 22.8 per 100 person-years for non-Type D and Type D persons, respectively [unadjusted hazard ratio (HR) 2.38, *p*=0.034]. In Cox regression analyses adjusted for age, gender, airway obstruction, and dyspnea, Type D personality was associated with increased risk for AECOPD (HR 2.63, *p*=0.046). The association diminished adding depression or anxiety to the model (HR 2.21, *p*=0.117 and HR 2.40, *p*=0.080, respectively). *Conclusion*: A minority of COPD patients has Type D personality, phenotypically recognized by a higher risk for psychological distress. These patients are more prone to AECOPD, but the importance of various psychological factors needs further studies.

Keyword: *COPD*

## 09

### Adherence and factors affecting satisfaction in long-term telerehabilitation for COPD patients

#### Hanne Hoaas ^1,2^, Linda Aaroen Lien^3^ and Paolo Zanaboni^2^

hanne.hoaas@telemed.no

^1^The Arctic University of Norway, Tromsoe, Norway; ^2^University Hospital of North Norway, Tromsø, Norway; ^3^LHL-klinikkene, Skibotn, Norway

#### 

*Introduction*: Telemedicine has the potential to increase accessibility and adherence to pulmonary rehabilitation in COPD. Adherence and satisfaction are important factors to consider before implementation in routine use. The study aimed to explore COPD patients’ experiences and adherence to long-term telerehabilitation in order to understand factors that affect satisfaction and identify elements for potential service improvement. *Methods*: A two-year pilot study with 10 patients with moderate or severe COPD was conducted. The intervention included treadmill exercise training at home, telemonitoring, and self-management via a webpage combined with weekly videoconference sessions with a physiotherapist. A qualitative study was based on two semi-structured focus group interviews and supplementary written questions. Data were analyzed by systematic text condensation. Adherence was measured in terms of dropouts and frequency of patients’ registrations of daily measurements and training sessions on the webpage. *Results*: Three major themes emerged regarding factors affecting satisfaction: (1) experienced health benefits, (2) empowerment, and (3) security in special competence and regular meetings. A fourth major theme was related to service improvement: (4) maintenance of motivation. No dropouts occurred. On average, patients registered 3.0 measurements/week and 1.7 training sessions/week. Frequency decreased from the first to the second year. *Conclusion*: The study shows that adherence to long-term telerehabilitation was supported by experienced health benefits and increased self-management in everyday life. Competent physiotherapists, who are able to empower the patients in their rehabilitation process, are considered crucial.

## 10

### High-sensitivity cardiac troponin T is associated with future exacerbations of COPD

#### Anke Neukamm ^1^, Vidar Søyseth^1^, Gunnar Einvik^1^, Nils Henrik Holmedahl^2^, Natalia Kononova^1^, Angelica Berg Gjørven^1^, Arne Didrik Høiseth^1^ and Torbjørn Omland^3^

anke.neukamm@medisin.uio.no

^1^Akershus University Hospital, Lørenskog, Norway; ^2^LHL-klinikkene, Norway; ^3^University of Oslo, Norway

#### 

*Introduction*: Cardiac troponin elevation, indicating myocardial injury, is associated with increased mortality in patients with acute exacerbation of chronic obstructive pulmonary disease (AECOPD). The prognostic value of circulating cardiac troponin T (cTnT) for future AECOPD hospitalizations among stable COPD patients has not been described previously. *Methods*: In a prospective cohort study we included 196 patients with stable COPD without prior diagnosis of cardiovascular disease from the Akershus University Hospital's outpatient clinic and from Glittreklinikken, a pulmonary rehabilitation clinic. cTnT – concentration was assessed with a high-sensitive assay, and characteristics of COPD-severity were registered at baseline. Subsequent hospitalization was recorded from hospital records (mean follow-up 2.7 years). *Results*: A total of 65 patients (33%) had at least one hospitalization for AECOPD. In total, 147 patients (75%) had measurable cTnT (>5 ng/L) and 49 patients (25%) had cTnT above the normal range (≥14.0 ng/L). Crude rates for AECOPD hospitalization were 7.0, 8.9, and 19.1 per 100 patient-years for patients with undetectable cTnT, cTnT in the normal range, and elevated cTnT, respectively. Adjusting for relevant covariates including lung function using Cox regression analysis, one ln increase of baseline cTnT was associated with a hazard ratio (95% confidence intervals) of 1.5 (1.1–2.0) for future AECOPD hospitalizations. *Conclusion*: cTnT elevation is frequently present in outpatients with stable COPD and is associated with increased risk for future hospitalization for AECOPD.

Keyword: *COPD*

## 11

### A mapping of personal factors and computer experience in people with chronic obstructive pulmonary disease involved in patient education at Karolinska University Hospital

#### Anna Karin Nordlin and Gun Faager
anki@nordlin.com
Karolinska Universitetssjukhuset, Stockholm, Sweden

#### 

*Introduction*: Health care providers are obliged to inform and make the patient involved through personalized information. To meet these requirements and use modern information technology, the physiotherapists at the Physiotherapy Clinic developed an interactive 3D visualization software for use in patient education for patients with chronic obstructive pulmonary disease (COPD). The survey should provide a picture of personal factors and computer skills of participants in the COPD program and provide a basis for the design, monitoring, and evaluation of interactive patient education. *Aim*: Identify the factors that may affect learning of patients participating in patient education. *Method*: Forty-seven patients with COPD participated in the study and underwent COPD program at the Karolinska University Hospital in Solna. Questionnaires on personal factors were answered on the basis of the following issues: habit in data technology profession and education, sense of coherence, SOC, the degree of anxiety and depression as measured by the Hospital Anxiety and Depression Scale (HADS), which learning style according to Kolb. *Results*: Forty-seven patients with COPD were included. The gender distribution of the group was 67% female and 33% male, and the average age of all 47 participants was 70 years (54–82). The results of this study are being processed and will be fully presented at the convention. *Conclusion*: A conclusion cannot be drawn since we have not processed all data.

Keywords: *COPD*; *rehabilitation*

## 12

### Diaphragm movements among patients with severe COPD and emphysema in standing forward leaning position with support of the forearms

#### Maria Ragnarsdottir ^1^, Hans Beck^2^, Magdalena Asgeirsdottir^2^, Petur Hannesson^1^, Skuli Oskar Kim^1^ and Asdis Kristjansdottir^2^

mariara@internet.is

^1^Landspitali National and University Hospital, Reykjavik, Iceland; ^2^Reykjalundur Rehabilitation Center, Iceland

#### 

*Introduction*: The position of diaphragm among patients with severe chronic obstructive pulmonary disease (COPD) and emphysema is lowered and thought to be more ineffective as hyperinflation increases. Consequently, it is common belief that these patients use thoracic-dominant breathing in this position especially with arms supported. *Objective*: To investigate respiratory movements in standing leaning forward position of patients with severe COPD and emphysema during rest and dyspnea. *Methods*: Eleven patients underwent spirometry measurement and positional assessment of diaphragm. Thoracic and abdominal respiratory movements during quiet breathing were measured in standing forward leaning position using the Respiratory Movement Measuring Instrument and simultaneously measuring diaphragmatic thickness with ultrasound imaging at three points along the zone of apposition during inspiration and expiration. Patients then bicycled with the work rate 87% of the peak work rate tolerated in a pre-program incremental exercise test. Immediately after dismounting the bicycle, respiratory movements were measured again in the same way and position. *Results*: Subjects mean age was 65±5 years, mean body mass index was 24.4±6.2, seven had GOLD 3 and four had GOLD 4. Mean dyspnea at rest was 0.9. Average range of abdominal movement was significantly (*p*>0.05) greater during dyspnea than at rest and average thoracic movement was significantly (*p>*0.05) less than abdominal. Diaphragm thickness significantly (*p*=0.02) increased in inspiration during dyspnea compared with at rest. *Conclusion*: This study indicates that contrary to general belief the diaphragm of patients with severe COPD and emphysema is active in forward leaning position with supported forearms during rest and dyspnea.

Keyword: *COPD*

## 13

### Results of an in-patient smoking cessation therapy provided as part of an interdisciplinary rehabilitation

#### Jonina Sigurgeirsdottir ^1^, Helga Jonsdottir^2^, Gudbjorg Petursdottir^1^ and Aldis Jonsdottir^1^

jonina@reykjalundur.is

^1^Reykjalundur Rehabilitation Centre, Mosfellsbær, Iceland; ^2^University of Iceland, Iceland

#### 

*Introduction*: Death rate from tobacco-related illness in Iceland is almost 400. Eliminating tobacco use is an important aim for tobacco users. Reykjalundur Rehabilitation Centre (RRC) has offered an interdisciplinary smoking cessation therapy since 1990. Current study compared results of a smoking cessation therapy for lung patients with other patient groups admitted to RRC. *Methods*: All smokers on the waiting list at RRC 2011–2013 were offered, without obligation, to participate in the study, which was approved by the Icelandic ethical authorities (*N*=120). Data were collected from January 1, 2011, and follow-up was completed in June 2014. Research questions: (1) What is the percentage of current smokers on admission abstinent to smoking and without relapse at 12 months post rehabilitation at RRC? (2) Is there a difference in smoking status between patients’ groups at 12 months post rehabilitation at RRC? SPSS was used for statistical tests with non-parametric and descriptive statistics with confidence limits at 95%. *Results*: A significant relationship was observed between diagnosis and abstinence without relapse at 12 months post rehabilitation and in reason for quitting tobacco use. Patients with lung diseases had highest quit rate (43.4%) than patients with pain or arthritis (25%); 7.1% of patients with heart/circulatory or CNS disease were smoke-free but no patients with psychiatric disease or obesity remained abstinent for 12 months. Patients with lung/heart/circulatory and CNS disease were more likely to quit because of physical illness than patients with arthritis/pain/psychiatric disease and obesity. *Conclusion*: The smoking cessation program at RRC is effective for patients with lung diseases and to an extent for patients with arthritis and pain diseases.

Keywords: *COPD*; *other*; *rehabilitation*

## 14

### Psychological distress and lung function in end-stage COPD patients

#### Torunn Stavnes Søyseth ^1^, Gro Killi Haugstad^2^, Øystein Bjørtuft^1^, May Britt Lund^1^, Aasta Heldal^1^, Vidar Søyseth^3^ and Ulrik Fredrik Malt^4^

tosoy@hotmail.com

^1^Oslo University Hospital, Oslo, Norway; ^2^Oslo and Akershus University College of Applied Sciences, Norway; ^3^Akershus University Hospital, Norway; ^4^University of Oslo, Norway

#### 

*Introduction*: Anxiety and depression may be serious comorbidities in patients with chronic obstructive pulmonary disease (COPD). The prevalence of anxiety and depression in COPD patients varies in different studies. The reasons may be use of different measuring instruments, and differences in populations. There is a need for more specific knowledge about psychological distress in COPD patients. *Aim*: To describe symptoms of psychological distress in end-stage COPD patients. *Method*: A total of 67 COPD patients [mean (SD) age 56 (5), 49% females] assessed for lung transplantation at Oslo University Hospital were enrolled in the study. All completed the questionnaires Hospital Anxiety and Depression Scale (HADS) and General Health Questionnaire-30 (GHQ-30). Spirometry and 6-min walk test were also performed. *Results*: 28% of the patients scored ≥8 on HADS anxiety and 21% scored ≥8 on HADS depression. Score ≥8 may be regarded as sign of psychological distress. In GHQ-30, total mean (SD) score was 29.1 (11.2). Score ≥30 indicates increased psychological distress. 29% of the patients expressed life as a struggle, 12% felt life was hopeless, while 20% experienced sleep disturbance caused by worries. There was significant correlation between psychological distress (GHQ-30 chronic score) and lung function (FEV1% predicted) (corr.coeff-0.3166, *p=*0.009). *Conclusion*: Many COPD patients suffer from psychological distress, experience life as a struggle, and have sleep disturbances. Correlation between psychological distress and lung function is statistically significant. These findings may have clinical implications.

Keyword: *COPD*

## 15

### Dyspnea – a frequent cause of hospital contacts: survey of a hospital in the region of southern Denmark

#### Ingrid Louise Titlestad, Zenel Demiri, Elias Choha and Annmarie T. Lassen
ingrid.titlestad@rsyd.dk
Odense University Hospital, Denmark

#### 

*Introduction*: We have performed retrospective audits on patients admitted with chronic obstructive pulmonary disease, where patients have been selected according to predefined discharge diagnoses (ICD-10). Until now, there has not been to our knowledge any systematic evaluation of patients presenting with respiratory symptoms (dyspnea) in emergency departments evaluating diagnoses, admission rate, or burden of multiple contacts. Since 2012 and reorganization of Emergency departments in Denmark, patients presenting with different symptoms trigger packages including predefined specific blood analyses and X-ray in order to secure efficient logistics and quicker evaluations, and ultimately avoid admissions. *Aims*: Course and diagnoses of patients presenting with dyspnea in a Danish hospital. *Methods*: Patient cohorts: all patients presenting with respiratory symptoms triggering a package (respiratory package and mostly presenting with dyspnea) were registered and retrieved for a period of 1 year (June 1, 2013 to May 31, 2014). The Danish Civil Registration enables the retrieval of full population-based data of all hospital contacts for all the registered outpatient contacts and admissions in the National Patient Register. Baseline data included gender, age, and number of contacts. Descriptive data as admission, registered ICD-10 diagnosis, and comorbidities were retrieved. *Results so far*: In one year, there were 3,017 contacts triggering a respiratory package, representing 2,183 unique patients. Further results and conclusions will be presented. *Perspectives*: The results will provide information on diversity of diagnoses and interestingly also on burden of contacts and constitute basis for more valid organizational decision making.

Keyword: *COPD*

## 16

### Validating the diagnosis exacerbation of chronic obstructive pulmonary disease in patients with very severe disease

#### Ulla Møller Weinreich ^1^, Jasmina Huremovic^1^, Ingolf Nissen^2^, Meika Helena Shakar^2,3^ and Line Hust Storgaard^1^

ulw@rn.dk

^1^Aalborg University Hospital; ^2^Sygehus Thy-Mors; ^3^Sygehus Vendsyssel, Denmark

#### 

*Introduction*: Chronic obstructive pulmonary disease (COPD) exacerbation (acute exacerbation of chronic obstructive pulmonary disease, AECOPD) is defined as ‘an acute event characterized by a worsening of the patient's respiratory symptoms that is beyond normal day-to-day variations and lead to a change in medication [Global Initiative for Chronic Obstructive Lung Disease (GOLD) 2013]’. Patients with very severe COPD often suffer from comorbidities that may cause dyspnea. This may influence the diagnosis AECOPD. This study investigates the inter-observer agreement of AECOPD diagnosis by three clinicians. *Method*: Twenty-two COPD patients, all treated with long-term oxygen therapy, were hospitalized 1–10 times over 6 months for AECOPD, in total 55 admissions. Retrospectively, three senior pulmonologists (P1, P2, and P3) evaluated the diagnosis as described by GOLD independently from the patients’ case files, blinded to treatment, and demographics. Events were classified by the majority opinion (2 out of 3). Results were analyzed and interpreted using Cohen's kappa coefficient and Landis-Koch's classification (<0 indicates no agreement, 0–0.20 slight agreement, 0.21–0.40 fair agreement, 0.41–0.60 moderate agreement, 0.61–0.80 substantial agreement, and 0.81–1 almost perfect agreement, assuming independence). *Results*: P1, P2, and P3 agreed on 43 out of 55 assessments. P3 agreed with the majority 11 times, more often than P2 (6) or P1 (7). Disagreement occurred 12 times. Heart disease was the most frequent diagnosis when disagreement occurred. *Conclusion*: The inter-observer agreement for verification of AECOPD was moderate to substantial with P3's opinion being most influential overall. AECOPD remains a difficult diagnosis.

**Table:** Results of the Kappa analysis

Assessment	Kappa
P1 vs. P2	0.49
P1 vs. P3	0.68
P2 vs. P3	0.68
Majority vs. P1	0.73
Majority vs. P2	0.73
Majority vs. P3	0.95

Keyword: *COPD*

## CANCER 17

### Prediction of pulmonary and non-pulmonary cancer incidence by level of emphysema and airway wall thickness in subjects with and without COPD

#### Ane Aamli ^1^, Miriam Gjerdevik^2^, Thomas Grydeland^1^, Frode Gallefoss^1^ and Per Bakke^1^

aneaamli@hotmail.com

^1^University of Bergen, Bergen, Norway; ^2^Haukeland University Hospital, Bergen, Norway

#### 

*Introduction*: There is limited knowledge of the prognostic value of quantitative computed tomography measures of emphysema and airway wall thickness on incidence of pulmonary and non-pulmonary cancer. We aim to examine this relationship, independent of smoking and age in subjects with and without COPD. *Methods*: We used data from a cohort of 886 ever-smokers, aged 40–75 years (49% with COPD), examined in 2003/04. The examination included CT thorax, lung function measurements, and questionnaires on smoking history. These data were merged with data from the Norwegian Cancer Register in a follow-up study over 8 years. Cox regression was used to compare those with medium degree of emphysema (3–10%) and those with high (above 10%) to those with less than 3% emphysema. *Results*: The hazard ratio for incidence of lung cancer for the medium group was 2.41 (95% CI: 0.70–8.30) and 7.34 (95% CI: 2.68–20.12) for the high group, compared to those with less than 3% emphysema. Hazard ratios for incidence of non-pulmonary cancer for the same groups were 1.08 (95% CI: 0.61–1.90) and 1.31 (95% CI: 0.77–2.20). Airway wall thickness did not predict pulmonary or non-pulmonary cancer independently. All were adjusted for sex, age, and several smoking variables. *Conclusion*: Level of emphysema assessed quantitatively from one CT scanner is independently related to pulmonary cancer, not to non-pulmonary cancer, in this population-based study of ever-smokers. Higher level of emphysema predicts larger incidence of lung cancer. Airway wall thickness is no predictor of cancer.

Keywords: *cancer*; *COPD*

## 18

### Is radial-EBUS with measurement of the distance between the orifice of bronchus and peripheral pulmonary lesions an effective diagnostic method of PPLs, of both benign and malign origin?

#### Amal Durakovic, Henrik Andersen and Irena Hammen
Amal.Durakovic@rsyd.dk
Odense University Hospital, Odense, Denmark

#### 

*Introduction*: Peripheral pulmonary lesions (PPLs) are known as a diagnostic challenge. The primary aim of this study was to investigate whether radial EBUS is an effective diagnostic method for PPLs, both benign and malign. *Methods*: This retrospective analysis of a database included all patients with PPLs undergoing radial EBUS from August 1, 2013 to August 31, 2014. The distance from the bronchial orifice to the pulmonary lesion was measured, and then the biopsy forceps were advanced to the measured distance with following transbronchial biopsy (TBB). *Results*: We examined 147 patients with PPLs using radial EBUS. Most of the lesions were between 21 and 30 mm (42%). The majority were located in the upper lobes (32% in the right and 26.5% in the left upper lobe). In total, 94 of 147 PPLs (63.9%) were detected by radial EBUS. In 34 cases (23%) fluorescence was additionally used to detect the lesion. A definitive diagnosis was established in 104 patients by TBB using radial EBUS (Efficacy of 70.7%). In 39 cases pathological tests proved malignancy [35 cases of lung cancer (LC) and four metastases from other cancers]. Benign pathological diagnoses included acute and chronic inflammation, and granulomas. The sensitivity of radial EBUS for the diagnostic of LC was 50.6 and 57.4% when the lesion was detected by radial EBUS. The specificity was on the other hand high (100%). The complication rate was low with three cases of pneumothorax. *Conclusion*: Radial EBUS with measurement of the distance between the orifice of bronchus and the lesion is an alternative diagnostic method of PPLs with a low complication rate. The diagnostic yield depends on the size and localization of the lesion. Yet, further method development is necessary in order to raise the diagnostic yield.

Keywords: *cancer*; *endoscopic intervention*

## 19

### The influence of demographic parameters and morbidity on hospitalization after lung cancer surgery

#### Asgerd Krogh Kristensen and Ulla Møller Weinreich
askk@rn.dk
Aalborg University Hospital, Denmark

#### 

*Introduction*: Lung cancer is one of the most common types of cancer in DK with about 4,300 new cases and 4,000 deaths every year. Patients are often aged, have reduced lung function, and suffer from comorbidities, and 20% of these patients may be candidates for surgery. This study investigates the influence of surgical intervention, demographic data, and comorbidities on length of stay (LOS) in hospital after lung resection. *Method*: A review of hospital records of 206 cancer patients who underwent either video-assisted thoracic surgery (VATS) or thoracotomy (TT) from January 2013 to September 2014 was performed. Age, gender, marital status (MS), lung function (LF), smoking status (SS), pack years (PY), body mass index (BMI), alcohol consumption (AC), number of comorbidities, Eastern Cooperative Oncology Group-score (ECOG), and operative method (OM) were registered. Subgroups operated with VATS and TT were analyzed via paired *T*-test. Pearson or Spearman's test were performed in the total population on LOS versus age; gender; MS; LF; SS; PY; BMI; AC; comorbidities ECOG and OM. Multiple regression analysis was performed including LOS versus LF, age, MS, BMI; ECOG (LOS vs. demographics) and LOS versus LF; SS; PY; AC; comorbidities and OM (LOS vs. morbidity). *Results*: There was no statistical difference between patients operated with VATS and TT. A non-significant correlation was seen between LOS and LF performing linear regression (*p*=0.07). Performing multiple regression analysis including LOS versus demographics *p*=0.01 for LF; between LOS versus morbidity *p*=0.04 for LF. *Conclusion*: There was no significant difference in LOS when comparing operation methods. LOS is associated with LF in multiple regression analysis, supported mainly by demographic parameters.

Keyword: *cancer*

## 20

### Patients' experience of their health changes when under investigation for pulmonary disease – the ongoing PEK-Lung project

#### Maria Olin ^1,2^, Bernhardson Britt-Marie^1^, Henoch Ingela^3^, Lindqvist Olav^1,4^, Rystedt Nadja^5^, Lehtiö Janne^1^, Pernemalm Maria^1^, Kölbeck Karl^1,2^, Tishelman Carol^1,2^ and Eriksson Lars E^1,6^

maria.olin@karolinska.se

^1^Karolinska Institutet, Stockholm, Sweden; ^2^Karolinska University Hospital Solna, Stockholm, Sweden; ^3^University of Gothenburg, Sweden; ^4^Umeå University, Sweden; ^5^Norrland's University Hospital, Umeå, Sweden; ^6^City University London, United Kingdom

#### 

*Introduction*: The majority of patients with lung cancer (LC) are diagnosed in advanced stages. Increased specificity about symptoms associated with LC might enable earlier diagnosis. This may allow timely alleviation of symptom distress and increase curative surgical treatment as an option. In this project, we study patients' subjective experiences of early health changes prior to LC diagnosis and link these changes to biomarker analyses. *Method*: Patients at initial evaluation at one ambulatory unit at Department of Respiratory Medicine, Karolinska University Hospital, Solna, Sweden, are asked to participate in the project. Patients complete an interactive individualized web-based questionnaire on a touchpad, consisting of 10 subject areas based on 163 descriptors generated through qualitative interviews. An assistant is available for further instructions or assistance. Blood samples are taken for the biobank. *Results*: From November 3, 2014, to March 1, 2015, 231 new patients were registered at the clinic, of which 195 were invited to participate (12 were excluded due to language difficulties and 24 cancelled their clinical appointment). In total, 122 (63%) of the patients fulfilling the inclusion criteria were included. Lack of time and fatigue were common reasons for non-participation. Our ambition is to include 500 patients. *Discussion*: This project is a unique collaboration, beginning with patient experiences, including research input spanning from bench to bedside, and driven by a nursing perspective. Challenges for feasibility, especially anchoring, ownership, and communication demands, will be discussed.

Keyword: *COPD*

## LABORATORY RESEARCH 21

### General gene expression analysis of human bronchial epithelial cells exposed to different concentrations of leukotriene E4

#### Siiri Altraja, Mario Mitt and Alan Altraja
siiri.altraja@ut.ee
University of Tartu, Estonia

#### 

The broader effect of leukotriene E4 (LTE4) on the gene expression in human bronchial epithelial cells (HBEC) is unknown. Cultured HBEC were exposed to 50, 100, and 250 nM LTE4 or media alone for 48 h. The total RNA (1 µg per sample) was used to prepare next-generation sequencing mRNA libraries, which were sequenced on Illumina HiSeq2500 platform. The results of meta-analysis of the differentially expressed genes in response to 50–250 nM LTE4 were subjected to pathway and network evaluation. Exposure to any concentration of LTE4 caused differential expression of 774 genes. Consistent changes by all concentrations of LTE4 used resulted in differential expression of 24 individual genes (*p*<0.05), 14 and 10 genes were up- and down-regulated, respectively. LTE4 significantly down-regulated genes that affect the biological pathways, including response to wounding, positive regulation of epithelial cell migration and osteoblast differentiation (e.g. protein kinase D1), epithelial fluid transport, and regulation of muscle adaptation (e.g. endothelin-1). The up-regulated pathways included dorsal/ventral pattern formation (e.g. inturned planar cell polarity protein). Both up- and down-regulated genes were affected in the muscle system process pathway. In conclusion, this study showed that the genes affected by LTE4 may substantially influence the epithelial response to the inflammatory injury and epithelial regeneration but also regulate the epithelial-mesenchymal transition as judged by the regulation of genes characteristic of muscle and other mesenchymal cells.

Supported by the Estonian Science Foundation grants No. 9103 and 9043.

Keyword: *laboratory research*

## 22

### Hypoxic pulmonary hypertension: a role for the inflammasome

#### Fadila Telarevic Cero, Karl-Otto Larsen, Vigdis Hilestad, Maria Belland Olsen, Camilla Udjus, Arne Yndestad, Pål Aukrust, Else Marit Løberg, Geir Christensen and Ole Henning Skjønsberg
cero.fadila@medisin.uio.no
Oslo University Hospital, Norway

#### 

*Introduction*: Patients with pulmonary hypertension have increased levels of interleukin (IL)-18 and IL-1β, and elevated levels of IL-18 have been found in alveolar hypoxia which leads to pulmonary hypertension. Caspase-1 cleaves IL-18 and IL-1β to their bioactive forms. Capase-1 is part of a large mulitprotein complex, also consisting of receptor (such as NLRP3) and the adaptor protein (ASC). Our objective was to study the role of the inflammasome components in chronic hypoxia-induced pulmonary hypertension. *Methods*: WT, NLRP3-/-, and ASC-/- mice were exposed to 3 months of hypoxia in a tightly sealed chamber containing 10% oxygen. Right ventricular systolic pressure (RVSP) was measured with a microtipped transducer catheter. To quantify muscularization and collagen content of pulmonary arteries, lung sections were stained with von Willebrand factor, smooth muscle α-actin, acid fuchsin orange G-stain (AFOG), and Sirius Red. *Results*: RVSP of NLRP3-/- exposed to hypoxia was not altered compared to WT hypoxia, while ASC-/- mice showed significantly lower RVSP than WT hypoxic mice, indicating reduced pulmonary hypertension. Hypoxic ASC-/- mice showed less degree of muscularization and fibrosis around pulmonary arteries compared to WT mice exposed to hypoxia. *Conclusions*: Depletion of the inflammasome adaptor ASC attenuates hypoxia-induced pulmonary hypertension, suggesting the inflammasome to be involved in development of pulmonary hypertension.

Keyword: *laboratory research*

## 23

### Clonality and transcriptional diversity of T-cell receptor-restricted CD4+ T cells in pulmonary sarcoidosis

#### Ylva Kaiser, Marcus Ronninger, Benita Dahlberg, Jan Wahlström, Anders Eklund and Johan Grunewald
ylva.kaiser@ki.se
Karolinska Institutet, Stockholm, Sweden

*Introduction*: Sarcoidosis is a granulomatous multisystem disorder of unknown etiology, characterized by accumulations of activated CD4+ T cells in the lungs. In bronchoalveolar lavage (BAL) fluid of patients positive for HLA DRB1*03, we have identified increased frequencies of CD4+ T cells expressing the T-cell receptor (TCR) chain combination Vα2.3 and Vβ22. These cells have a higher activation state than autologous Vα2.3− Vβ22− T cells, indicating an effector function in pulmonary antigen recognition. *Aim*: Phenotypic and functional characterization of the CD4+ T-cell population in sarcoidosis, particularly in the context of Vα2.3+ Vβ22+ T cells. *Methods*: Lineage-specific surface markers, transcription factors, and proliferative capacity of BAL cells were assessed by flow cytometry and clonality through next-generation sequencing. *Results*: In all sarcoidosis patients, CD4+ T-cell proliferation was increased in BAL compared to blood. Intriguingly, co-expression of Th1 and Th17 transcriptional regulators T-bet and RORyT was observed in a majority of BAL lymphocytes but not in blood. Vα2.3+ Vβ22+ T cells expressed both transcription factors, potentially reflecting more potent immune recognition and antigen clearance. Also, a more pronounced ratio of T-bet+ RORyT+ versus T-bet single-positive cells in Löfgren's syndrome (LS) patients than in non-LS points to an association between IL-17 and a good prognosis. Vα2.3 and Vβ22 TCR sequencing revealed a high degree of clonality, in accordance with antigen recognition. *Conclusion*: The CD4+ T-cell profile in sarcoidosis is likely more complex than previously described. Co-expression of key regulatory elements and clonal expansion of Vα2.3+ Vβ22+ T cells might be essential for a successful antigen-specific response.

Keywords: *interstitial lung disease*; *laboratory research*

## OTHER 24

### Resistance patterns in multidrug-resistant tuberculosis in Estonia

#### Alan Altraja ^1^, Piret Viiklepp^2^, Manfred Danilovits^3^ and Lea Pehme^3^

alan.altraja@kliinikum.ee

^1^University of Tartu, Tartu, Estonia; ^2^National Institute for Health Development, Tallinn, Estonia; ^3^Tartu University Hospital, Estonia

#### 

*Introduction*: Multidrug-resistant tuberculosis (MDR-TB) accounts for 19.6% of the pulmonary culture-positive newly detected cases in 2014. However, a detailed overview of the particular resistance patterns present in the MDR-TB cases enabling optimization of the case management is missing. *Objective*: To determine the particular patterns of drug resistance in MDR-TB cases diagnosed in Estonia. *Methods*: A country-wide retrospective survey was conducted to analyze resistance to all first- (FLD) and second-line drugs (SLD) in the strains isolated from new pulmonary MDR-TB cases registered at the Estonian TB Registry 2012–2014. Hierarchical cluster analysis using between-group linkage method was performed. *Results*: Of the isolates from 97 patients, 43 (56.2%) were resistant to all five first-line drugs. The full resistance profiles for SLD and FLD other than RMP and INH were available for 90 cases. Of these, 4 cases (4.4%) were defined as XDR-TB and 30 (33.3%) as pre-XDR-TB. Fifteen different patterns of drug resistance were found. Simultaneous resistance to INH, RMP, SM, and EMB accounted for 27 (30.0%) of cases, followed by co-resistance to INH, RMP, SM, EMB, and PZN in 20.0%. Less frequent co-resistance patterns were INH, RMP, SM, EMB, PZN, and OF (8.9%), INH, RMP, SM, EMB, and KA (8.9%) and INH, RMP, SM, EMB PZN, OF, and PT (3.3%). According to the resistance, the drugs were subdivided into two clusters containing pure FLD in one and pure SLD in the other. *Conclusion*: MDR-TB treatment should be started before having all susceptibility results, preferably without FLD. The complicated resistance pattern urges use of new drugs for treatment of M/XDR TB in Estonia to improve the treatment outcome.

Keyword: *other*

## 25

### Extrinsic allergic alveolitis against goose down feather as occupational disease

#### Ulrich Mack ^1^, Britt G. Randem^2^, Carl Birger Alm^1^ and Sjur Humerfelt^3^

dr.mack.ulrich@gmail.com

^1^Lovisenberg Diakonale Sykehus, Oslo, Norway; ^2^Oslo University Hospital, Norway; ^3^KAL klinikken, Oslo, Norway

#### 

A 39-year-old male presented with an 8 months history of cough and dyspnea on exertion. Lung function tests revealed a severe restriction with forced vital capacity (FVC) 2.04 L (36%), and DLCO was reduced to 30.6%. High-resolution CT scan of the lungs showed widespread small nodular ill-defined changes with centrilobular distribution typical for EAA. Transbronchial lung biopsy was described as fibrosis and chronic inflammation, CD4/CD8 ratio was 1.15. The patient has a positive antibody pattern consistent with M. Sjögren, but the lung lesions were considered not be related to this. There was a positive IgG antibody titer against *Aspergillus fumigatus* (76 mg/L), but when the patient's office in an unventilated basement room was checked, no mold fungi antigens were found. The patient is working as purchasing manager for a sports outfitter chain with special responsibility for down garments. A new laboratory test (Institut für Prävention und Arbeitsmedizin, Bochum) showed an IgG titer of 31.6 mgA/L against goose feather antigens, besides being positive against *A. fumigatus* and several bird species. The patients lung function improved greatly with steroid treatment of initially 1 mg/kg daily, but remained restrictive with an FVC of 3.76 L (67%) and TLC 6.38 L (79%). DLCO had improved to 60.3%. On an excursion involving spending the night outdoors using a down sleeping bag, he had a relapse with general malaise due to influenza-like symptoms. We referred the patient for occupational medicine evaluation, and the patients’ disease was reported to the appropriate authorities as occupational disease; recognition is pending. Feather duvet lung is a known disease entity, but to our knowledge never described as occupational disease triggered by down garments outside of the production process.

Keywords: *asthma/allergy*; *interstitial lung disease*; *occupational/environmental medicine*

